# USP10 inhibits the degradation of α-synuclein, a pathogenic factor associated with Parkinson's disease, by inhibiting chaperone-mediated autophagy

**DOI:** 10.1016/j.jbc.2025.110292

**Published:** 2025-05-24

**Authors:** Sergei Anisimov, Masahiko Takahashi, Taichi Kakihana, Yoshinori Katsuragi, Junya Sango, Takayuki Abe, Masahiro Fujii

**Affiliations:** Division of Virology, Niigata University Graduate School of Medical and Dental Sciences, Niigata, Japan

**Keywords:** USP10, α-synuclein, Parkinson’s disease, сhaperone-mediated autophagy

## Abstract

Parkinson's disease (PD) is a progressive neurodegenerative disorder characterized by loss of dopaminergic neurons, particularly in the substantia nigra of the brain. **α**-Synuclein is a major causative factor in both familial and sporadic forms of PD, and its protein aggregates play critical roles in neuronal cell death and PD pathogenesis. This study explored the role of ubiquitin-specific protease 10 (USP10) in the regulation of **α**-synuclein in neuronal cells. Knockdown of USP10 in SH-SY5Y neuronal cells led to a reduction in **α**-synuclein levels, which was reversed by inhibiting chaperone-mediated autophagy (CMA) through lysosome-associated membrane protein 2A depletion, a protein essential for CMA. A novel CMA reporter with a specific CMA degradation motif further demonstrated that knockdown of USP10 activated CMA in neuronal cells. In addition, USP10 overexpression increased the levels of both WT and five PD-associated **α**-synuclein mutants, whereas a deubiquitinase-deficient USP10 mutant did not increase **α**-synuclein levels. This study provides new insights into the mechanisms that regulate **α**-synuclein proteostasis and highlights USP10 as a promising drug target for PD.

Parkinson’s disease (PD) is a progressive neurodegenerative disorder characterized by loss of dopaminergic neurons, particularly in the substantia nigra of the brain (1, 2). PD affects approximately 1% of individuals older than 60 years, making it one of the most prevalent neurodegenerative diseases (3). A hallmark of PD pathogenesis is the accumulation and aggregation of α-synuclein, forming neurotoxic α-synuclein fibrils that contributes to dopaminergic neuronal death (4). Genetic evidence further supports the critical role of α-synuclein in PD, as both duplication and triplication of the α-synuclein gene cause familial PD, with triplication leading to more severe disease progression than duplication (5). This gene dosage effect implies that elevated α-synuclein levels accelerate α-synuclein aggregation, fueling disease progression. Given the central role of α-synuclein in PD pathology, it has become a prime therapeutic target, with strategies aimed at reducing its levels or preventing its aggregation, efforts that are considered a promising approach to slow or potentially halt PD progression.

Recent research has highlighted the importance of cellular α-synuclein clearance mechanisms in PD pathology, particularly the roles of autophagy and the ubiquitin–proteasome system. Chaperone-mediated autophagy (CMA) is a key cellular mechanism for α-synuclein degradation (6, 7). CMA selectively degrades proteins containing KFERQ or KFERQ-like peptides, which are also present in α-synuclein (8). These CMA substrates bind to heat shock cognate protein 70 (HSC70) chaperone proteins *via* KFERQ-like motifs and are transported to lysosomes, where CMA substrates interact with the lysosomal membrane protein lysosome-associated membrane protein 2A (LAMP2A) and move into the lysosomal lumen for degradation by lysosomal enzymes. The inhibition of CMA has been shown to increase the amount and toxicity of α-synuclein in cells and exacerbate PD-like phenotypes in animal models (9, 10). Furthermore, accumulating evidence suggests that the CMA is impaired in the brains of patients with PD (11). In addition, CMA reduces the amount and toxicity of proteins associated with neurodegenerative diseases beyond α-synuclein, such as tau protein in Alzheimer's disease (AD) (12) or Huntingtin protein in Huntington's disease (13).

Recent studies have implicated ubiquitin-specific protease 10 (USP10), a deubiquitinating enzyme, in PD pathogenesis. USP10 is ubiquitously expressed in various cell types, including neurons (14). Our previous studies suggested that USP10 promotes α-synuclein aggregation during PD development. For instance, USP10 colocalizes with Lewy bodies (LBs) in the brains of patients with PD and promotes α-synuclein-containing protein aggregates (called aggresomes) with properties similar to LBs in cells treated with proteasome inhibitors, and USP10 overexpression increases α-synuclein levels in cultured non-neuronal cells (15). Furthermore, USP10 inhibits apoptosis in dopaminergic neuron–like cell lines treated with dopamine in a manner dependent on the reduction in reactive oxygen species (16).

In the present study, we demonstrated that USP10 increased α-synuclein abundance in neuronal cells by inhibiting CMA. Our findings show that the deubiquitinase activity of USP10 is essential for its role in promoting α-synuclein accumulation and that pharmacological inhibition of USP10 leads to α-synuclein reduction. These results indicate that USP10 promotes pathogenic α-synuclein aggregation in PD, making USP10 inhibition a promising therapeutic target for preventing α-synuclein accumulation in PD.

## Results

### USP10 depletion reduces endogenous **α**-synuclein levels in a neuronal cell line

**Figure 1:**
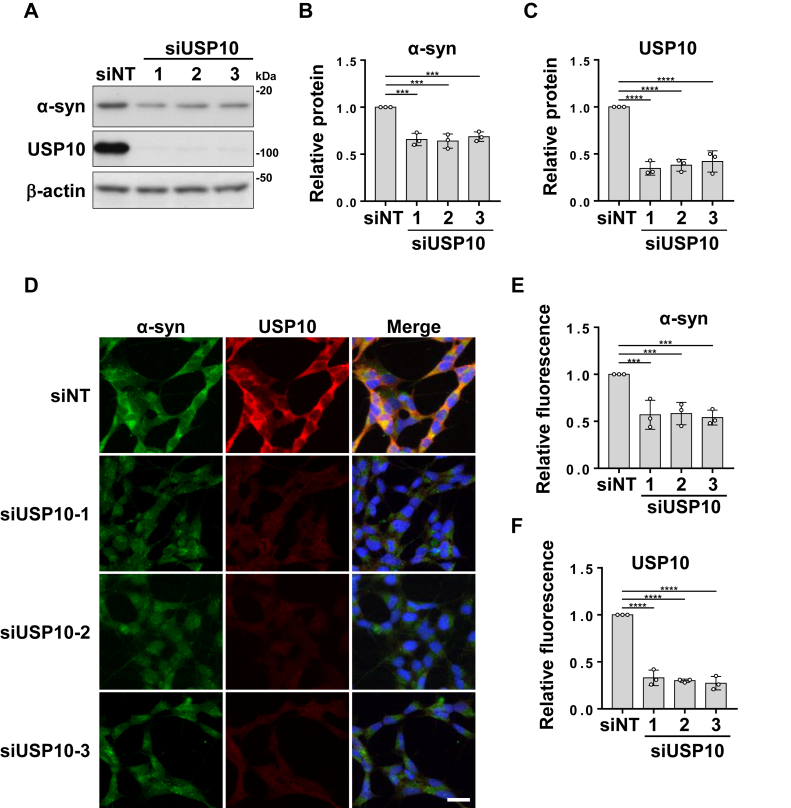
**Reduction of USP10 decreases endogenous α-synuclein (α-syn) levels in cells.***A*–*C*, SH-SY5Y cells were transfected with three different USP10-siRNAs (siUSP10) or nontargeting siRNA (siNT) using Lipofectamine RNAiMAX. Whole-cell lysates were analyzed by Western blotting using anti-α-syn, anti-USP10, and anti-β-actin antibodies. The ratio of the α-syn band to the β-actin band was measured by densitometry, and the mean and SD from three experiments are presented in *B* and *C*, respectively. The significance of the differences was assessed by a one-way ANOVA followed by Dunnett's multiple comparisons test. ∗∗∗*p* < 0.001; ∗∗∗∗*p* < 0.0001. *D*–*F*, SH-SY5Y cells were grown on coverslips, transfected with three different siUSP10 or siNT, and stained with anti-α-syn (*green*) and anti-USP10 (*red*) antibodies, whereas nuclei were stained with Hoechst 33258 (*blue*). The fluorescence intensity of α-syn (*E*) and USP10 (*F*) was measured by fluorescence microscopy, and the ratio of intensity for each knockdown sample compared with the control was calculated from 100 cells and presented as mean ± SD. The significance of the differences was assessed by a one-way ANOVA followed by Dunnett's multiple comparisons test. ∗∗∗*p* < 0.001; ∗∗∗∗*p* < 0.0001. Scale bars represent 20 μm. USP10, ubiquitin-specific protease 10.

SH-SY5Y is a dopaminergic neuron–like cell line. To investigate the role of USP10 in regulating α-synuclein protein levels in neuronal cells, we knocked down USP10 expression in SH-SY5Y cells using three different siRNAs (Fig. 1*A*). A Western blot analysis revealed that all three USP10 knockdown (USP10-KD) lines showed significantly reduced α-synuclein protein levels in SH-SY5Y cells compared with the control (Fig. 1, *A*–*C*). Similarly, an immunofluorescence analysis showed decreased α-synuclein staining in USP10-KD SH-SY5Y cells (Fig. 1, *D*–*F*). These findings suggest that a decrease in USP10 protein levels leads to a decrease in α-synuclein protein levels in neuronal SH-SY5Y cells at steady state.

### Depletion of USP10 decreases **α**-synuclein levels *via* the autophagy pathway

**Figure 2:**
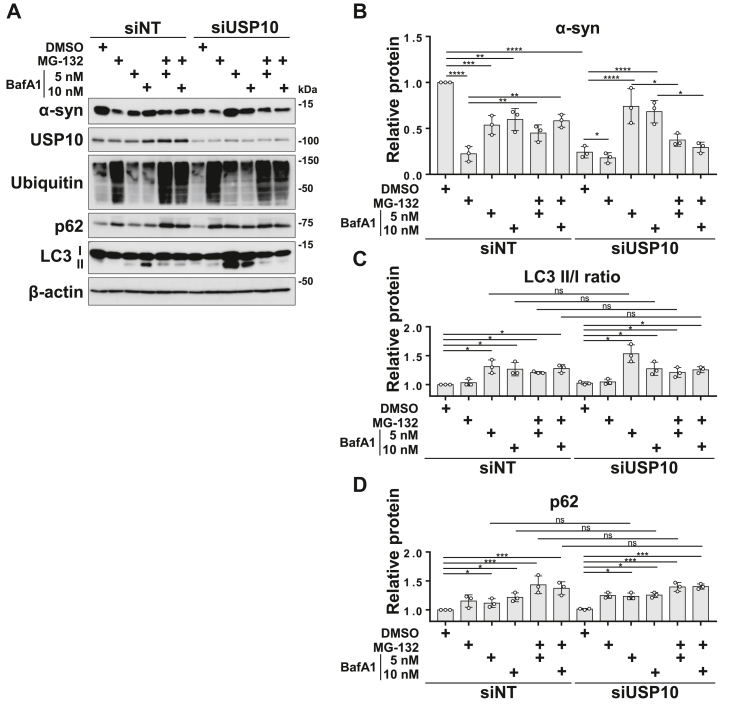
**Inhibition of autophagy suppresses degradation of α-synuclein (α-syn) by USP10-KD.***A*–*D*, SH-SY5Y cells were transfected with siUSP10 or a nontargeting siRNA, followed by the addition of 5 μM MG-132, 5 or 10 nM bafilomycin A1 for 12 h prior to harvest. Whole-cell lysates were analyzed by Western blotting using anti-α-syn, antiubiquitin, anti-p62, anti-LC3, anti-USP10, and anti-β-actin antibodies. The amount of the α-syn and p62, and ratios of LC3-II/LC-I bands to the β-actin were measured by densitometry, and the mean and SD from three experiments are presented in *B*–*D*. The significance of the differences was assessed by a one-way ANOVA followed by Tukey's multiple comparisons test. ns, not significant, ∗*p* < 0.05; ∗∗*p* < 0.01; ∗∗∗*p* < 0.001; and ∗∗∗∗*p* < 0.0001. USP10-KD, ubiquitin-specific protease 10 knockdown.

To investigate the involvement of autophagy or proteasomes in the reduction of α-synuclein by USP10-KD, USP10-KD SH-SY5Y cells were treated with the lysosomal inhibitor bafilomycin A1 (BafA1) to inhibit autophagy and/or the proteasome inhibitor MG-132 for 12 h. The reduction of α-synuclein by USP10-KD was reversed by the inhibition of autophagy (BafA1) to the level observed in WT cells treated with BafA1 (Fig. 2, *A* and *B*). In contrast, MG-132 reduced the amount of α-synuclein in USP10 WT cells, and this reduction was restored by BafA1 treatment (Fig. 2, *A* and *B*), indicating that MG-132-induced reduction of α-synuclein was due to autophagy triggered by proteasome inhibition by MG-132, as reported in previous studies (17, 18). The increase in LC3-II/LC3-I ratio and p62 by BafA1 and the increase in ubiquitin levels by MG-132 confirmed that BafA1 and MG-132 inhibited autophagy and proteasomes, respectively. In addition, our analysis showed that USP10-KD in SH-SY5Y cells enhances α-synuclein degradation without altering LC3-II/LC3-I ratios or p62 levels, excluding LC3/p62-dependent autophagy activity in USP10-KD–induced α-synuclein degradation (Fig. 2, *C* and *D*). Taken together, these findings suggest that USP10-KD enhances α-synuclein degradation in SH-SY5Y cells through LC3/p62-independent autophagy pathways, such as CMA, rather than LC3-dependent macroautophagy.

### USP10-KD activates CMA to degrade α-synuclein

**Figure 3:**
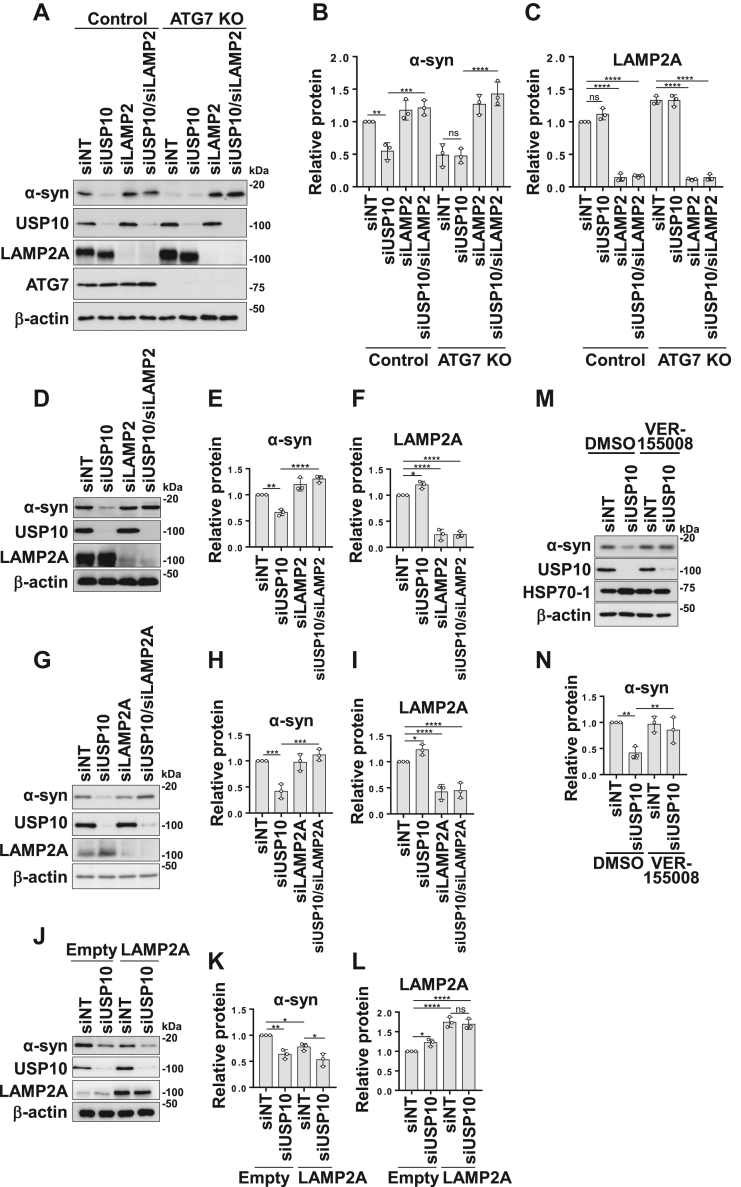
**USP10-KD induces degradation of α-synuclein (α-syn) by CMA.***A*–*C*, ATG7 KO or control HeLa cells were transfected with siRNA targeting USP10, LAMP2, or their combination (siUSP10/siLAMP2), or a nontargeting siRNA. Whole-cell lysates were analyzed by Western blotting using anti-α-syn, anti-USP10, anti-LAMP2A, anti-ATG7, and anti-β-actin antibodies. The ratios of the α-synn and LAMP2A bands to the β-actin band were measured by densitometry, and the means and SD from three experiments are presented in *B* and *C*. The significance of the differences was assessed by a one-way ANOVA followed by Tukey's multiple comparisons test. ∗∗*p* < 0.01; ∗∗∗*p* < 0.001; and ∗∗∗∗*p* < 0.0001; ns, not significant. *D*–*F*, SH-SY5Y cells were transfected with siRNA targeting LAMP2, USP10, or a control siRNA. Whole-cell lysates were analyzed by Western blotting using anti-α-syn, anti-LAMP2A, anti-USP10, and anti-β-actin antibodies. The ratios of the α-syn and LAMP2A bands to the β-actin band were measured by densitometry, and the mean and SD from three experiments are presented in *E* and *F*, respectively. The significance of the differences was assessed by a one-way ANOVA followed by Tukey's multiple comparisons test. ∗*p* < 0.05; ∗∗*p* < 0.01; and ∗∗∗∗*p* < 0.0001. *G*–*I*, SH-SY5Y cells were transfected with siRNA against LAMP2A, USP10, or control nontargeting siRNA. Whole-cell lysates were characterized by Western blot analysis using anti-α-syn, anti-LAMP2A, anti-USP10, and anti-β-actin antibodies. The ratio of the α-syn or LAMP2A bands relative to the β-actin bands was measured by densitometry scanning, and the mean and SD from three experiments are presented in *H* and *I*, respectively. The significance of the differences was assessed by a one-way ANOVA followed by Tukey's multiple comparisons test. ∗*p* < 0.05; ∗∗*p* < 0.001; and ∗∗∗∗*p* < 0.0001. *J*–*L*, 293T cells were transfected with USP10 siRNA or control nontargeting siRNA, with or without the LAMP2A expression plasmid. Whole-cell lysates were characterized by Western blot analysis using anti-α-syn, anti-LAMP2A, anti-USP10, and anti-β-actin antibodies. The ratio of the α-syn or LAMP2A bands relative to the β-actin bands was measured by densitometry scanning, and the mean and SD from three experiments are presented in *K* and *L*, respectively. The significance of the differences was assessed by a one-way ANOVA followed by Tukey's multiple comparisons test. ns, nonsignificant, ∗*p* < 0.05; ∗∗*p* < 0.01; and ∗∗∗∗*p* < 0.0001. *M* and *N*, SH-SY5Y cells were transfected with USP10 siRNA or a nontargeting siRNA. Prior to harvesting, the cells were treated with 25 μM VER-155008 for 24 h. Whole-cell lysates were analyzed by Western blotting using anti-α-syn, anti-USP10, anti-HSP70-1, and anti-β-actin antibodies. The ratio of α-syn bands to β-actin bands was measured by densitometry, and the means and SD from three experiments are presented at *N*. The significance of the differences was assessed by a one-way ANOVA followed by Tukey's multiple comparisons test. ∗∗*p* < 0.01. USP10-KD, ubiquitin-specific protease 10 knockdown. CMA, chaperone-mediated autophagy; LAMP2A, lysosome-associated membrane protein 2A; USP10-KD, ubiquitin-specific protease 10 knockdown.

Macroautophagy and CMA are the two main autophagy pathways involved in α-synuclein degradation, with CMA playing a more prominent role at steady state (6). Therefore, we investigated whether macroautophagy or CMA was involved in the degradation of α-synuclein in USP10-KD HeLa cells. LAMP2A is a critical factor for CMA but not macroautophagy (19). Knockdown of LAMP2A using LAMP2 siRNA (LAMP2-KD) fully restored α-synuclein levels in USP10-KD cells (Fig. 3, *A*–*C*). These results indicated that CMA is responsible for the degradation of α-synuclein in USP10-KD HeLa cells.

The ATG7 gene function is essential for macroautophagy but not for CMA. To investigate the role of macroautophagy in α-synuclein degradation by USP10-KD, we used HeLa cells with a knockout of the ATG7 gene (ATG7-KO) and further knocked down LAMP2. The α-synuclein protein level was lower in ATG7-KO cells than in ATG7 WT cells, but these lower α-synuclein levels were restored by LAMP2 knockdown (Fig. 3, *A*–*C*). These results indicate that α-synuclein was degraded by CMA in ATG7-KO cells. Previous studies have also shown that inhibition of macroautophagy by ATG7-KO activates CMA, thereby reducing α-synuclein levels (20). Interestingly, USP10-KD did not further reduce α-synuclein levels in ATG7-KO cells, suggesting that USP10-KD may induce CMA *via* a mechanism similar to that of ATG7-KO (Fig. 3, *A*–*C*). Taken together, these results suggest that USP10-KD promoted α-synuclein degradation by activating CMA.

We also examined the role of LAMP2A in the USP10-KD–induced reduction of α-synuclein in SH-SY5Y cells (Fig. 3*D*). Similar to what was observed in HeLa cells, depletion of LAMP2A by LAMP2 siRNA in SH-SY5Y cells restored the reduction in α-synuclein, indicating that USP10-KD reduces the amount of α-synuclein by activating CMA in neuronal SH-SY5Y cells (Fig. 3, *D*–*F*). Because there are three isoforms of LAMP2 (LAMP2A, LAMP2B, and LAMP2C) produced from the same gene by alternative splicing, and each product has different activities, with only LAMP2A regulating CMA, we also used specific siRNAs that target only LAMP2A. LAMP2A-KD also rescued the amount of α-synuclein in USP10-KD cells similar to LAMP2-KD, confirming LAMP2A-dependent CMA in USP10-KD cells (Fig. 3, *G*–*I*). Interestingly, the amount of LAMP2A was significantly increased by USP10-KD in SH-SY5Y cells (Fig. 3, *F*–*I*). To explore the role of elevated LAMP2A expression in the degradation of α-synuclein by USP10-KD, we overexpressed LAMP2A in 293T cells (Fig. 3*J*). LAMP2A overexpression reduced α-synuclein levels, consistent with its role in CMA, as shown in a previous report (21). Notably, USP10-KD combined with LAMP2A overexpression further diminished α-synuclein levels beyond LAMP2A overexpression alone (Fig. 3, *J*–*L*). These results suggest that USP10-KD induces α-synuclein degradation by increasing the amount of LAMP2A in SH-SY5Y cells, but additional activities of USP10-KD also induce α-synuclein degradation.

HSC70 is a chaperone essential for CMA, as it binds to CMA substrates and transports them to lysosomes. VER-155008 (VER) is an inhibitor of heat shock response 70 (HSP70) family members, including HSC70. VER inhibited the USP10-KD–induced reduction of α-synuclein (Fig. 3, *M* and *N*), suggesting that USP10-KD promotes α-synuclein degradation by activating HSC70-dependent CMA. Interestingly, USP10-KD increased the amount of HSP70-1, another family member of HSP70, and this increase was reversed by VER (Fig. 3, *M* and *N*). HSP70-1 promotes α-synuclein degradation *via* both the ubiquitin–proteasome system and autophagy pathways, including CMA (22). Therefore, the elevated levels of HSP70-1 observed in USP10-KD cells may further contribute to the reduction of α-synuclein in USP10-KD cells.

### Depletion of USP10 induces the perinuclear localization of lysosomes

**Figure 4:**
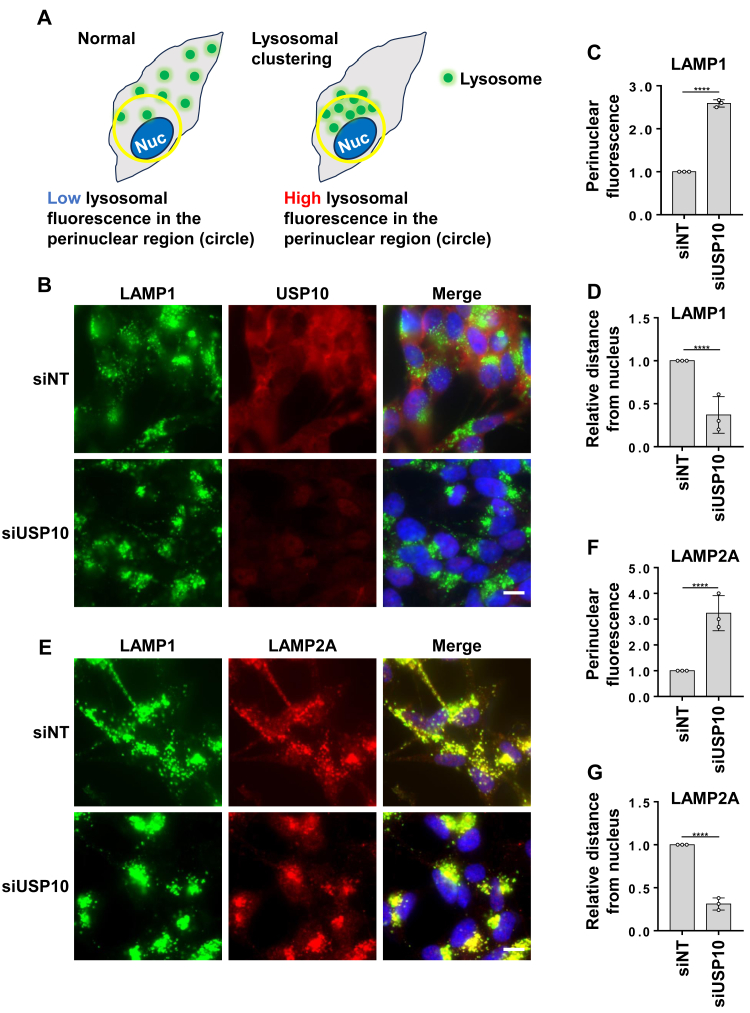
**Perinuclear clustering of LAMP2A-positive lysosomes in USP10-KD cells.***A*, a schematic representation of perinuclear lysosome measurement. *B*–*D*, SH-SY5Y cells grown on cover slips were transfected with siRNA targeting USP10 or a control siRNA and subsequently stained with anti-LAMP1 (*green*) and anti-USP10 (*red*) antibodies, followed by staining with Hoechst 33258 (*blue*). The perinuclear localization of lysosomes was analyzed by measuring the fluorescence intensity around the center of the nucleus compared with the intensity near the cell border (*C*) and by assessing the relative proximity of lysosome dots to the nuclear border (*D*) in USP10-KD cells compared with control cells. The significance of the differences was assessed using unpaired two-sample *t* test. ∗∗*p* < 0.01; ∗∗∗*p* < 0.001. Scale bars represent 10 μm. *E*–*G*, SH-SY5Y cells grown on coverslips were transfected with siRNA targeting USP10 and stained with anti-LAMP1 (*green*) and anti-LAMP2 (*red*) antibodies, and nuclei were stained with Hoechst 33258 (*blue*). The perinuclear localization of lysosomes was analyzed by measuring the fluorescence intensity around the center of the nucleus compared with the intensity near the cell border (*F*) and by assessing the relative proximity of lysosome dots to the nuclear border (*G*) in USP10-KD cells compared with control cells. The significance of the differences was assessed using unpaired two-sample *t* test.∗∗*p* < 0.01; ∗∗∗*p* < 0.001. Scale bars represent 10 μm. LAMP2A, lysosome-associated membrane protein 2A; USP10-KD, ubiquitin-specific protease 10 knockdown.

Recent studies have underscored the importance of lysosomal distribution in the regulation of autophagy. Previous reports indicate that CMA-activating stimuli, such as nutrient starvation, induce perinuclear localization of lysosomes near microtubule-organizing centers (20, 23). Such lysosomal clustering in the perinuclear region has been shown to enhance CMA-mediated lysosomal degradation of CMA substrates (24). Therefore, we investigated the subcellular localization of lysosomes in USP10-KD cells (Fig. 4*A*). The depletion of USP10 by siRNA in SH-SY5Y cells induced clustered lysosomes in the perinuclear region stained by lysosome marker LAMP1 (Fig. 4, *B*–*D*). In addition, USP10-KD increased LAMP2A staining in the perinuclear region (Fig. 4, *E*–*G*). The perinuclear localization and clustering of LAMP2A-positive lysosomes in USP10-KD cells further supports the activation of CMA by USP10 depletion.

### A novel CMA reporter detects USP10-KD–induced CMA activation

**Figure 5:**
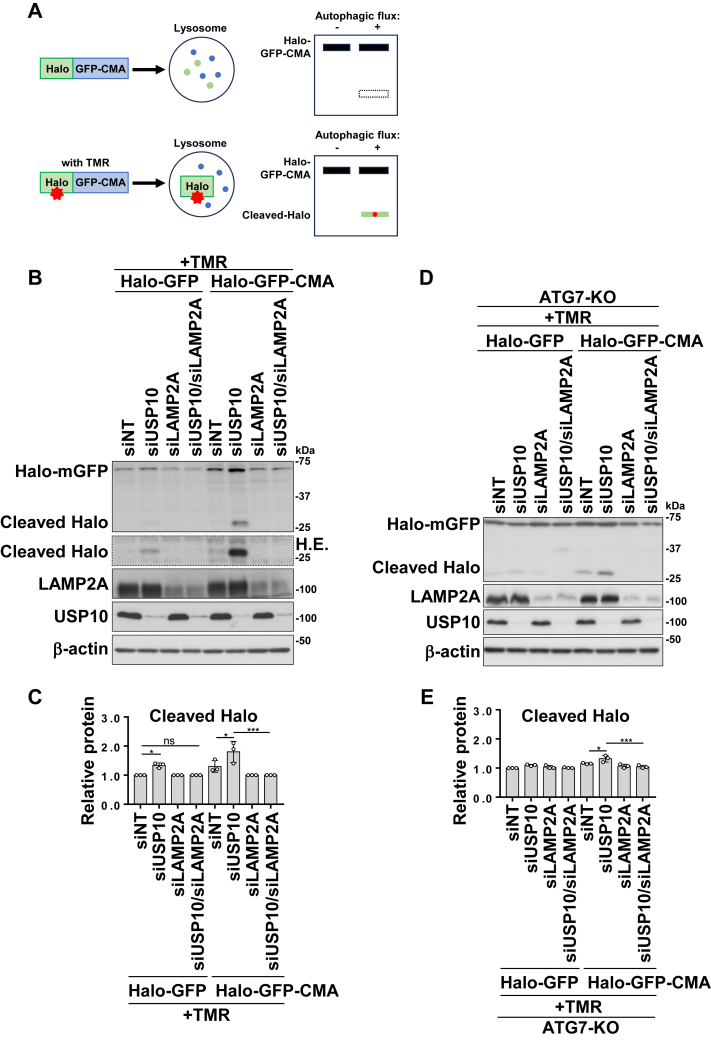
**USP10-KD increase degradation of CMA-targeted probe.***A*, schematic of the CMA degradation probe experiment. The Halo-tag was fused to a GFP protein containing the CMA motif. Upon addition of the TMR ligand, Halo-tag became resistant to lysosomal degradation, allowing measurement by Western blotting. *B* and *C*, SH-SY5Y cells expressing Halo-GFP or Halo-GFP-CMA were transfected with siRNA against LAMP2A, USP10, their combination (USP10/LAMP2A), or a nontargeting siRNA. Cells were incubated with the TMR ligand for 20 min at 24 h prior to collection, and whole-cell lysates were prepared. Western blot analysis was performed using anti-Halo-tag, anti-LAMP2A, anti-USP10, and anti-β-actin antibodies. The ratio of cleaved Halo bands relative to β-actin bands was measured by densitometry scanning, and the mean and SD from three experiments are presented in *C*. The significance of the differences was assessed by a one-way ANOVA followed by Tukey's multiple comparisons test. ∗*p* < 0.05; ∗∗*p* < 0.01; ns, not significant. *D* and *E*, the Halo-GFP or Halo-GFP-CMA plasmids was transfected into ATG7-KO HeLa cells together with LAMP2A siRNA, USP10 siRNA, or a combination of the two (USP10/LAMP2A). Cells were incubated with the TMR ligand for 20 min 24 h prior to cell collection, and whole-cell lysates were prepared. Western blot analysis was performed using anti-Halo-tag, anti-LAMP2A, anti-USP10, and anti-β-actin antibodies. The ratio of cleaved Halo bands relative to β-actin bands was measured by densitometry scanning, and the mean and SD from three experiments are presented in *E*. The significance of the differences was assessed by a one-way ANOVA followed by Tukey's multiple comparisons test. ∗*p* < 0.05; ∗∗∗*p* < 0.001. CMA, chaperone-mediated autophagy; LAMP2A, lysosome-associated membrane protein 2A; USP10-KD, ubiquitin-specific protease 10 knockdown.

To assess CMA activity in an alternative manner, we constructed a novel CMA reporter that fuses a Halo-tagged GFP with a CMA-targeting motif (Halo-GFP-CMA) based on a recently developed method to measure autophagy (25). We mutated the C-terminal amino acid sequence of the GFP protein to create a KFERQ-like peptide sequence that functions as a CMA degradation motif and induces CMA-mediated degradation of the Halo-GFP-CMA protein. The Halo tag acts as a receptor and binds to the cell-permeable fluorescent probe TMR. When TMR binds, the Halo–TMR complex of Halo-GFP-CMA is stabilized, and only GFP is degraded by lysosomes. Accumulated Halo fragments generated from Halo-GFP-CMA were detected using Western blotting. The CMA-specific degradation of Halo-GFP-CMA was examined by comparing it to that of Halo-GFP (Fig. 5*A*). To investigate CMA activity regulated by USP10, we established SH-SY5Y cell lines stably expressing either Halo-GFP or Halo-GFP-CMA. Subsequently, the cells were transfected with siRNAs targeting USP10 and LAMP2A. USP10-KD increased the amount of cleaved Halo, which was reduced by codepletion of LAMP2A (Fig. 5, *B* and *C*). These results indicate that Halo-GFP-CMA detected USP10-KD–induced CMA in SH-SY5Y cells. In addition, USP10-KD increased the amount of Halo fragments from GFP-Halo lacking the CMA motif to a much lesser extent, and this amount was also reduced by LAMP2A-KD. The weak degradation of GFP-Halo by USP10-KD may be explained by the weak cryptic CMA motif in GFP-Halo. Next, to investigate the mechanism by which USP10-KD activates the Halo-GFP-CMA probe, we investigated the activity of USP10-KD in the macroautophagy-deficient ATG7-KO HeLa cells expressing Halo-GFP-CMA. USP10-KD increased the amount of Halo fragments cleaved from Halo-GFP-CMA, and this amount was reduced by LAMP2A-KD (Fig. 5, *D* and *E*). On the other hand, USP10-KD little induced Halo fragments from Halo-GFP. These results suggest that USP10-KD activates CMA even in the macroautophagy-deficient ATG7-KO cells. In addition, since USP10-KD does not reduce the amount of α-synuclein in ATG7-KO cells, the detection sensitivity of USP10-KD activity against CMA probe appears to be higher than USP10-KD activity against α-synuclein.

### USP10 increases the amount of mutant **α**-synuclein in SH-SY5Y cells

**Figure 6:**
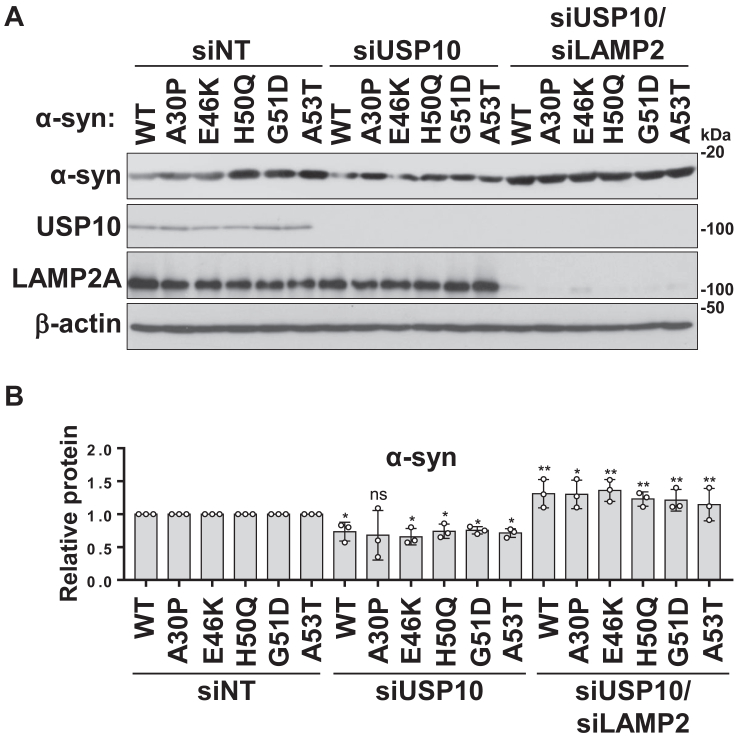
**USP10-KD promotes CMA degradation of WT α-synuclein (α-syn) and Parkinson's disease–associated α-syn mutants in SH-SY5Y cells.***A* and *B*, SH-SY5Y cells stably expressing WT or mutant α-syn were transfected with USP10 siRNA, a combination of USP10 siRNA and LAMP2 siRNA (siUSP10/siLAMP2), or nontargeting siRNA. Whole-cell lysates were analyzed by Western blotting using anti-α-syn, anti-USP10, anti-LAMP2A, and anti-β-actin antibodies. The ratio of α-syn bands relative to β-actin bands was measured by densitometry scanning, with the means and SD from three experiments presented in *B*. The significance of the differences was assessed by a one-way ANOVA followed by Tukey's multiple comparisons test. ∗*p* < 0.05; ∗∗*p* < 0.01; ns, not significant. CMA, chaperone-mediated autophagy; LAMP2A, lysosome-associated membrane protein 2A; USP10-KD, ubiquitin-specific protease 10 knockdown.

Mutations in α-synuclein have been linked to familial PD. To investigate whether USP10 is involved in the stability of mutant α-synuclein proteins, we established SH-SY5Y cells stably expressing WT or five disease-associated α-synuclein mutants: A30P, E46K, H50Q, G51D, and A53T. USP10-KD significantly reduced the amount of α-synuclein in WT and four of the five mutant cell lines (excluding A30P), and this reduction was reversed by the addition of LAMP2-KD (Fig. 6, *A* and *B*). The amount of A30P was also reduced by USP10-KD, but this reduction was not statistically significant. These results suggest that USP10 inhibits the degradation of α-synuclein by CMA in SH-SY5Y cells, with or without α-synuclein mutations.

**Figure 7:**
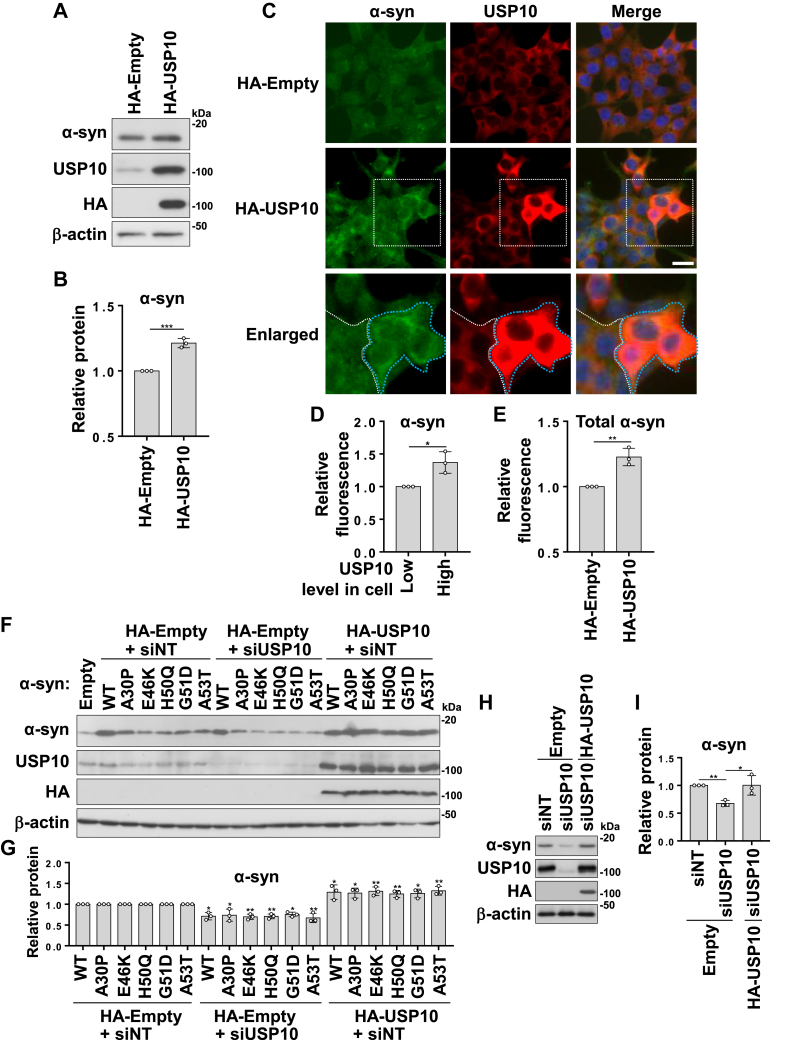
**Overexpression of USP10 increases α-synuclein (α-syn) levels in 293T cells.***A* and *B*, 293T cells were transfected with HA-USP10 or HA-empty plasmid. Whole-cell lysates were analyzed by Western blotting using anti-α-syn, anti-USP10, anti-HA, and anti-β-actin antibodies. The ratio of α-syn bands relative to β-actin bands was measured by densitometry scanning, with means and SD from three experiments presented in *B*. The significance of the differences was assessed using an unpaired two-sample *t* test. ∗∗∗*p* < 0.001. *C*–*E*, 293T cells were transfected with HA-USP10 or HA-empty plasmid and stained with anti-α-syn (*green*) and anti-USP10 (*red*) antibodies; nuclei were stained with Hoechst 33258 (*blue*). The total fluorescence intensity of the α-syn signal was measured at *D*, whereas the level of α-syn in cells with high USP10 expression is shown in *E*. The significance of the differences was assessed using unpaired two-sample *t* test. The bars represent 20 μm; ∗*p* < 0.05; ∗∗*p* < 0.01. *F* and *G*, 293T cells were transfected with WT α-syn or its mutants (A53E, E46K, H50Q, G51D, and A53T), in combination with HA-USP10 or an HA-empty plasmid. In addition, cells were transfected with either nontargeting siRNA or USP10 siRNA. Whole-cell lysates were analyzed by Western blotting using anti-α-syn, anti-USP10, anti-HA, and anti-β-actin antibodies. The ratio of α-syn bands relative to β-actin bands was measured by densitometry scanning, and the means and SD from three experiments are presented in *G*. The significance of the differences was assessed by a one-way ANOVA followed by Dunnett's multiple comparisons test. ∗*p* < 0.05; ∗∗*p* < 0.01. *H* and *I*, 293T cells were transfected with nontargeting siRNA or siRNA targeting the 3′-UTR of USP10 mRNA together with HA-USP10 with the deletion of 3′-UTR or HA-empty plasmid. The whole-cell lysates were analyzed by Western blotting using anti-α-syn, anti-USP10, anti-HA, and anti-β-actin antibodies. The ratio of α-syn bands relative to β-actin bands was measured by densitometry scanning, and the means and SD from three experiments are presented in *I*. The significance of the differences was assessed by a one-way ANOVA followed by Tukey's multiple comparisons test. ∗*p* < 0.05; ∗∗*p* < 0.01. HA, hemagglutinin; USP10, ubiquitin-specific protease 10.

In addition, transient USP10 overexpression in 293T cells significantly increased α-synuclein levels (Fig. 7, *A* and *B*). An immunofluorescence analysis revealed that α-synuclein levels increased in cells overexpressing USP10 (Fig. 7, *C* and *D*). Notably, the signal intensity of α-synuclein was increased in cells with high USP10 expression (Fig. 7*C*, *enlarged image* and 7E). These findings indicate that elevated USP10 levels lead to increased intracellular α-synuclein levels. Overexpression of PD-associated α-synuclein mutants also increased α-synuclein levels to the same extent as WT α-synuclein, suggesting that USP10 inhibits the degradation of WT and mutant α-synuclein with similar efficiency (Fig. 7, *F* and *G*). Transfection with the siRNA-resistant USP10 plasmid restored the reduced amount of α-synuclein in USP10-KD cells, further confirming the role of USP10 in α-synuclein expression (Fig. 7, *H* and *I*).

### USP10 increases the amount of **α**-synuclein in a manner dependent on deubiquitinase activity

**Figure 8:**
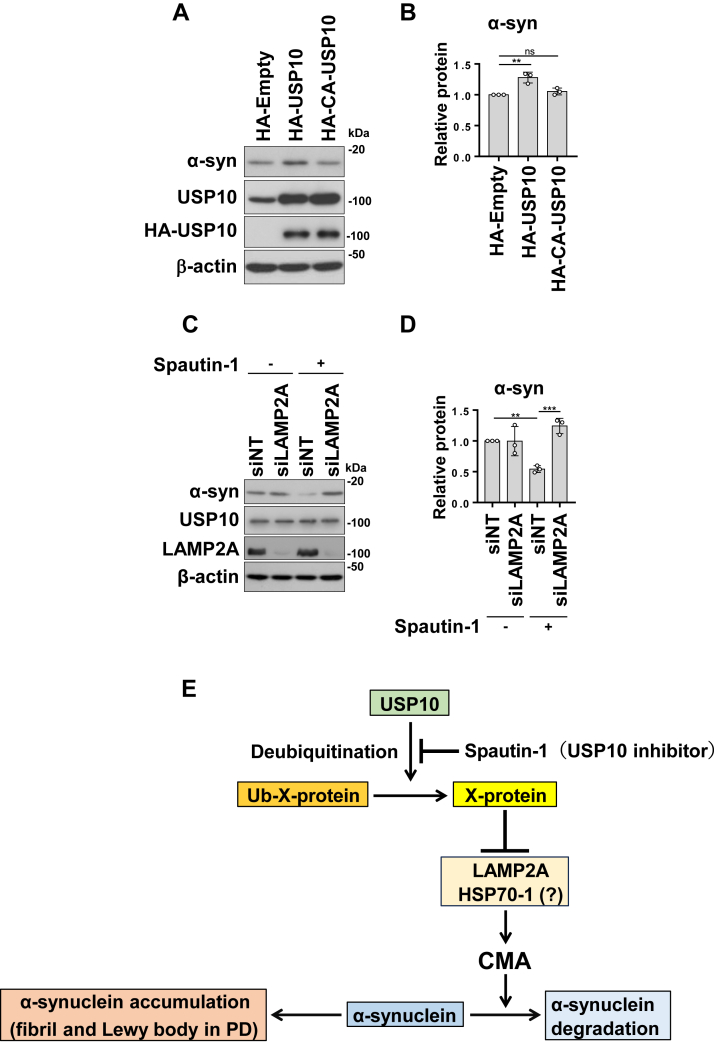
**Spautin-1 treatment decreases α-synuclein (α-syn) degradation in LAMP2A-dependent manner.***A* and *B*, 293T cells were transfected with HA-USP10, HA-CA-XUSP10, or HA-empty plasmid. Whole-cell lysates were analyzed by Western blotting using anti-α-syn, anti-USP10, anti-HA, and anti-β-actin antibodies. The ratio of α-syn bands relative to β-actin bands was measured by densitometry scanning, and the mean and SD from three experiments are presented in *B*. The significance of the differences was assessed by a one-way ANOVA followed by Tukey's multiple comparisons test. ∗∗*p* < 0.01; ns, not significant. *C* and *D*, SH-SY5Y cells were transfected with siRNA targeting LAMP2A and treated with 10 μM Spautin-1 for 36 h. Whole-cell lysates were analyzed by Western blotting using anti-α-syn, anti-LAMP2A, anti-USP10, and anti-β-actin antibodies. The ratio of α-syn bands relative to β-actin bands was measured by densitometry scanning, and the mean and SD from three experiments are presented in *D*. The significance of the differences was assessed by a one-way ANOVA followed by Tukey's multiple comparisons test. ∗∗*p* < 0.01; ∗∗∗*p* < 0.001. *E*, proposed research model. USP10 removes ubiquitin molecules from protein X. The deubiquitinated protein X suppresses CMA by reducing the amount of LAMP2A and HSP70-1 and increases the amount of α-syn. Abnormal inhibition of CMA promotes the accumulation and aggregation of α-syn, the formation of α-syn fibril/oligomer and Lewy bodies, and α-syn fibril/oligomer are involved in the onset and progression of PD. Conversely, the inhibition of USP10 activity by Spautin-1 increases ubiquitinated protein X, increases CMA activity, and promotes the degradation of α-syn. This cascade ultimately reduces the level of α-syn and reduces the aggregation of α-syn. CMA, chaperone-mediated autophagy; HA, hemagglutinin; HSP70, heat shock response 70; LAMP2A, lysosome-associated membrane protein 2A; PD, Parkinson's disease; USP10, ubiquitin-specific protease 10.

USP10 has deubiquitinase activity, which deubiquitinates multiple substrate proteins (14). Unlike WT USP10, transient overexpression of the USP10 mutant (CA-USP10) did not increase α-synuclein levels (Fig. 8, *A* and *B*). These results indicated that USP10 increased the amount of α-synuclein in a deubiquitinase-dependent manner by inhibiting CMA.

Spautin-1 is a chemical compound that inhibits the deubiquitinase activity of USP10. The treatment of SH-SY5Y cells with Spautin-1 for 36 h reduced the amount of α-synuclein, and this reduction was abolished by LAMP2A-KD (Fig. 8, *C* and *D*). These results also indicate that the deubiquitinase activity of USP10 is required for the increase in α-synuclein levels induced by USP10 in SH-SY5Y cells.

## Discussion

Our study demonstrated that USP10 promotes α-synuclein accumulation in neuronal cells by inhibiting CMA (Fig. 8*E*). The role of CMA dysfunction in PD pathogenesis has gained recognition in recent years (26). For instance, PD-associated α-synuclein mutants have been shown to inhibit CMA, facilitating the accumulation and aggregation of mutant proteins (27). In addition, the familial PD-related mutant LRRK2 (G2019S) obstructs CMA activity by preventing multimerization of LAMP2A at the lysosomal membrane (28). Our findings expand this body of research by identifying USP10 as an additional inhibitor of CMA that contributes to α-synuclein accumulation. Notably, we observed that the pharmacological inhibition of USP10 using Spautin-1 led to a reduction in α-synuclein levels (Fig. 8*C*). This suggests that targeting USP10 to enhance CMA activity represents a promising therapeutic strategy for PD and other diseases associated with CMA dysfunction.

Our findings showed that USP10-KD led to the accumulation and clustering of lysosomes in the perinuclear cytoplasmic region of cells (Fig. 4). Perinuclear localization of lysosomes is a hallmark of enhanced CMA activity. Previous studies have shown that various stimuli, such as macroautophagy inhibition, serum starvation, and other CMA activators, can induce lysosomal repositioning. Importantly, perinuclear lysosomes typically exhibit higher CMA activity than those located peripherally (24). Therefore, our results suggest that USP10-KD stimulates CMA by promoting perinuclear accumulation of lysosomes. However, the results of this study do not rule out the possibility that the accumulation of lysosomes in the perinuclear region is not the cause of CMA activation by USP10-KD but rather a result of CMA activation by USP10-KD.

Our study suggests that the mechanism by which USP10-KD activates CMA involves the increased expression of LAMP2A and HSP70-1 (Fig. 3, *D*–*N*). USP10-KD significantly increased the expression of LAMP2A in SH-SY5Y cells (Fig. 3, *C*–*F*). Furthermore, overexpression of LAMP2A in 293T cells reduced α-synuclein levels (Fig. 3, *J*–*L*). LAMP2A is a well-established rate-limiting factor for CMA, and its overexpression enhances CMA activity and is reported to promote α-synuclein degradation (29). Therefore, the upregulation of LAMP2A in SH-SY5Y cells following USP10-KD likely contributed to the observed activation of CMA and the subsequent reduction in α-synuclein levels. Interestingly, the impact of USP10-KD on LAMP2A expression appears to be cell type specific. Unlike SH-SY5Y cells, HeLa cells did not show a significant increase in LAMP2A levels after USP10-KD (Fig. 3*A*). This discrepancy suggests that the mechanisms by which USP10-KD activates CMA vary among different cell types.

In addition to LAMP2A, we observed an increase in HSP70-1 protein levels following USP10-KD (Fig. 3, *M* and *N*). HSP70-1 is a multifunctional chaperone protein that is known to enhance various protein degradation systems, including macroautophagy and the ubiquitin–proteasome system (22). It directly interacts with α-synuclein, facilitating its clearance and preventing its accumulation and aggregation (30). The increase in HSP70-1 levels following USP10-KD may represent another mechanism by which USP10-KD activates CMA. For example, HSP70-1 may facilitate the transport of CMA substrates to lysosomes (29), thereby promoting CMA activity. This suggests multiple mechanisms by which USP10 controls CMA activity under various conditions.

Our study indicated that the deubiquitinase activity of USP10 is essential for its inhibitory effect on CMA (Fig. 8, *A*–*D*). Previous research has shown that USP10 activates macroautophagy by deubiquitinating several substrates, including Beclin-1, a key initiator of the macroautophagic pathway (31). This finding raises the possibility that USP10 inhibits CMA through the activation of macroautophagy *via* deubiquitination. Our data, however, suggest that this mechanism does not underlie CMA regulation by USP10-KD. Although USP10-KD sufficiently reduced α-synuclein, there was no significant change in the LC3-II/LC-I ratio, indicating that macroautophagy activity is not involved in the degradation of α-synuclein by USP10-KD. Furthermore, USP10-KD enhanced CMA in ATG7-KO cells, which lack functional macroautophagy (Fig. 5*D*). Since the amount of α-synuclein in ATG7-KO cells is not further reduced by USP10-KD, it is possible that the CMA-dependent degradation of α-synuclein caused by the inhibition of macroautophagy in ATG7-KO cells masks the α-synuclein degradation activity of USP10-KD. These findings contradict a macroautophagy-dependent activity in USP10-KD–induced CMA activation and instead imply that USP10 may directly deubiquitinate CMA-inhibitory/stimulatory proteins, modulating their stability or function (Fig. 8*E*).

Interestingly, USP10-KD and USP10 overexpression similarly regulated the expression of WT α-synuclein and five PD-associated α-synuclein mutants (Figs. 6, *A* and *B* and 7, *F* and *G*). In contrast, several previous studies have shown that CMA is less effective at degrading several α-synuclein mutants than WT α-synuclein (32). Although the reasons for this discrepancy are unclear, they may stem from differences in the experimental conditions of the studies. For instance, CMA activity varies across tissues and is higher in the liver, kidney, and spleen than in other organs. In addition, various factors may regulate CMA differently in different cell types and tissues.

In PD, α-synuclein forms toxic oligomers and fibrils, and these α-synuclein oligomers/fibrils are the main cause of the death of dopaminergic neurons in PD (33). LBs are a pathological feature of PD, and their main component is α-synuclein. Interestingly, recent studies have shown that the toxic effects of α-synuclein oligomers and fibrils are lost when they are sequestered in LBs, suggesting that LBs may reduce the toxic effects of α-synuclein oligomers and fibrils (34). Our previous studies showed that, under stress conditions, cultured cells form protein aggregates called aggresomes that contain α-synuclein, and that aggresome formation is involved in the suppression of cell death caused by stress (15). Importantly, USP10 increases the amount of α-synuclein in cultured cells and promotes the formation of α-synuclein-positive aggresomes (15). Aggresomes have been shown to be structurally and biochemically similar to LBs (35). Therefore, USP10 may reduce the toxicity of α-synuclein oligomers and fibrils by forming LBs in PD. In this study, we further extended these results and suggested that USP10 promotes the aggregation of α-synuclein by inhibiting the degradation of α-synuclein by CMA. Therefore, USP10 appears to have a dual effect on the amount, aggregation, and toxicity of α-synuclein in PD. First, USP10 increases the amount of α-synuclein by inhibiting its CMA-mediated degradation. Second, USP10 may induce the formation of LBs that reduce the toxic effects of α-synuclein oligomers/fibrils. These two actions of USP10 on α-synuclein may be important at different stages of the development and progression of PD.

Tau and TDP-43 are causative factors for AD and amyotrophic lateral sclerosis, respectively. In addition to α-synuclein, USP10 increases the amount of tau and TDP-43 and induces the formation of protein aggregates (15). Similar to α-synuclein, both tau and TDP-43 are substrates of CMA (12, 36). Therefore, investigating the role of USP10-regulated CMA in protein aggregation and pathogenesis of AD and amyotrophic lateral sclerosis could provide valuable insights. Furthermore, since CMA has been implicated in the degradation of various disease-associated proteins beyond neurodegenerative diseases, targeting USP10-regulated CMA represents a promising therapeutic strategy for treating these conditions, including cancer.

Our Halo-GFP-CMA reporter demonstrated sufficient sensitivity for detecting CMA induction, as evidenced by its ability to capture CMA activation triggered by USP10-KD. The Halo-GFP-CMA reporter system is promising for applications in cells and potentially in animal models as well. This versatility opens new avenues for understanding the role of CMA in various cellular processes and disease states.

## Experimental procedures

### Cell lines and culture condition

SH-SY5Y cells, a human neuroblastoma cell line, were cultured in Dulbecco's modified Eagle's medium (DMEM) supplemented with 5% heat-inactivated fetal bovine serum, Opti-MEM (Thermo Fisher Scientific), 4 mM l-glutamine 50 units/ml penicillin, 50 μg/ml streptomycin, and MEM Nonessential AA solution. HeLa, 293T, and Plat-E cells were cultured in DMEM supplemented with 10% heat-inactivated fetal bovine serum 4 mM l-glutamine, 50 units/ml penicillin, 50 μg/ml streptomycin, and MEM nonessential amino acid solution (Thermo Fisher Scientific).

#### Reagents and antibodies

The following reagents were purchased from the indicated companies for use in this study: MG-132 (474790; Calbiochem), Hoechst 33258 (H-3569; Molecular Probes), Spautin-1 (567569; Sigma–Aldrich), BafA1 (B1793; Sigma–Aldrich), blasticidin (R21001; Thermo Fisher Scientific), and VER (SML0271-5MGM; Merck). The following antibodies were used in this study: anti-USP10 (A300-901A; Bethyl Laboratories; HPA006731; Sigma–Aldrich), antiubiquitin (sc-8017; Santa Cruz Biotechnology), anti-p62 (PM045; MBL), anti-GFP (sc-9996; Santa Cruz Biotechnology), anti-α-synuclein (S5566; Sigma–Aldrich), anti-Halo-tag (G921; Promega), anti-β-actin (sc-47778; Santa Cruz Biotechnology), anti-hemagglutinin (HA) (2367S; Cell Signaling Technology), anti-ATG7 (#8558; Cell Signaling Technology), anti-LAMP2A (ab18528; Abcam), anti-LAMP1 (ab24170; Abcam), anti-HSP70 (AD1-SPA-810; Enzo Life Sciences), and anti-LC3 (PM036B; MBL).

#### Plasmids

Expression plasmids encoding HA-tagged USP10 (HA-USP10), deubiquitinase-dead-USP10 mutant (CA-USP10), CSII-CMV-Syn-IRES2-Bsd, pCMV-VSV-G-RSV-Rev, and pCAG-HIVgp have been previously described (15). pMD.G is the expression vector for the envelope glycoprotein (G protein) of vesicular stomatitis virus and was kindly provided by Dr Didier Trono (Swiss Federal Institute of Technology in Lausanne, Switzerland). pMRX-IB-HaloTag7-mGFP was obtained from Addgene (#184903). pMRX-IB-HaloTag7-mGFP-KFERQ was generated in our laboratory, as described later. Plasmids encoding α-synuclein mutants (A30P, E46K, H50Q, G51D, or A53T) were created from the pcDNA-α-synuclein-WT plasmid (a gift from Dr Hasegawa) by introducing point mutations using the PrimeSTAR basal mutagenesis kit (R046A; TAKARA). A nontagged LAMP2A expression plasmid vector was obtained from OriGene (#324903).

#### Plasmid transfection

HeLa and 293T cells (1.5 × 10^5^) were seeded onto a 6-well plate (Corning) the day before transfection. Cells were then transfected with the plasmid using FuGENE 6 in Opti-MEM (1869048; Life Technologies) according to the manufacturer’s instructions (Roche). The cells were harvested for analysis 24 h after transfection.

#### RNA interference

siRNAs specific to human USP10 (Oligo IDs: HSS113446, HSS113447, and HSS113448) and negative control siRNA (catalog no.: 12935-100) were purchased from Thermo Fisher Scientific. The siRNA (SI02669121) targeting the 3′-UTR of USP10 mRNA and the siRNA (SI02780638) targeting LAMP2 mRNA were obtained from QIAGEN. Custom sequence siRNA against LAMP2A was ordered from Eurofins (GCACCAUCAUGCUGGAUAUAU/AUAUAGGCUGCAUGACCAUG). siRNAs were transfected into cells using Lipofectamine RNAiMAX reagent, according to the manufacturer’s protocol (Thermo Fisher Scientific).

### Western blot analyses

Cells were lysed with SDS lysis buffer (62.5 mM Tris–HCl, pH 6.8, 2% SDS, 10% glycerol, 5% 2-mercaptoethanol, and 0.005% bromophenol blue), and cell lysates (20 μg) were separated by SDS-PAGE and electrophoretically transferred onto a polyvinylidene difluoride (PVDF) membrane (Immobilon; Millipore). PVDF membranes were incubated with 5% skimmed milk and further treated with the indicated antibodies diluted in Can Get (TOYOBO). Immunoreactive bands were detected using an enhanced chemiluminescence (ECL) detection system (ECL Western Blotting Detection Reagents; GE Healthcare; Pierce ECL Plus Western Blotting Substrate; Thermo Fisher Scientific) and visualized using Amersham Hyperfilm ECL (GE Healthcare). For the detection of α-synuclein protein, the PVDF membrane was fixed with 0.4% paraformaldehyde before incubation with skimmed milk, since α-synuclein protein is easily separated from the membrane (37).

#### Establishment of stable synuclein-expressing SH-SY5Y cell lines

To produce lentiviruses expressing human α-synuclein and its mutants, PlatE cells were transfected with the CSII-CMV-MCS-IRES2-Bsd lentivirus vector encoding nontagged WT α-synuclein or its point mutants (A30P, E46K, H50Q, G51D, or A53T), along with pCMV-VSV-G-RSV-Rev and pCAG-HIVgp, using FuGENE 6, according to the manufacturer’s instructions. At 72 h post-transfection, the culture supernatant containing the viruses was collected and used to infect SH-SY5Y cells in the presence of 8 μg/ml polybrene. At 48 h after infection, the cells were cultured in medium containing 7 μg/ml blasticidin. The expression of α-synuclein in SH-SY5Y cells was evaluated using Western blotting.

### Immunofluorescence analyses

Cells were plated on glass coverslips in a 6-well plate and fixed with 3.7% formaldehyde in PBS at room temperature for 15 min. Subsequently, cells were permeabilized with 0.1% Triton X-100 in PBS at room temperature for 5 min. The fixed cells were incubated with the primary antibody at room temperature for 60 min, washed with PBS, and then incubated with the secondary antibody and Hoechst 33258 for nuclear staining at room temperature for an additional 60 min. Fluorescence microscopy was performed using a BZ-8000 microscope (KEYENCE), and an image analysis was conducted using the BZ-II analyzer software program (KEYENCE). The secondary antibodies used were anti-mouse immunoglobulin labeled with either Alexa 488 or Alexa 594 and anti-rabbit immunoglobulin labeled with either Alexa 488 or Alexa 594 (Molecular Probes). The expression of proteins in the cells was quantified using the BZ-II analyzer, and the fluorescence intensity was presented as the ratio of total intensities. The perinuclear localization of lysosomes was analyzed by measuring the fluorescence intensity around the center of the nucleus compared with the intensity near the cell border. In addition, the relative proximity of lysosomal dots to the nuclear border was measured in USP10-KD cells compared with control cells. Data on perinuclear distribution were obtained from more than 100 cells across the three coverslips. Cell counts were determined using Hoechst staining.

#### CMA reporter experiment

pMRX-IB-HaloTag7-mGFP is a retroviral expression plasmid encoding Halo-GFP (Addgene #184903) (25). The pMRX-IB-HaloTag7-mGFP-KFERQ plasmid, which encodes Halo-GFP-CMA, was created by mutating the C terminus of mGFP in pMRX-IB-HaloTag7-mGFP to a KFERQ-like motif (QELFK) using the PrimeSTAR basal mutagenesis kit (R046A; TAKARA) and the following primer sets: forward primer (GGACCAGGAGCTGTTCAAGTAACTCGAGAGC) and reverse primer (AACAGCTCCTGGTCCATGCCGAGAGTGATCC). To generate retroviruses expressing Halo-GFP and Halo-GFP-CMA, pMRX-IB-HaloTag7-mGFP or pMRX-IB-HaloTag7-mGFP-KFERQ plasmids were transfected into retrovirus packaging PlatE cells along with pMD.G plasmids. At 72 h post-transfection, the culture supernatants containing the retroviruses were collected and used to infect SH-SY5Y cells in the presence of 8 μg/μl polybrene. At 48 h after infection, the cells were cultured in selection medium containing blasticidin. To assess the degradation of Halo-GFP or Halo-GFP-CMA *via* CMA, SH-SY5Y cells stably expressing either Halo-GFP or Halo-GFP-CMA were incubated in DMEM with 100 nM TMR-conjugated Halo ligand (G8251; Promega) for 20 min, followed by two washes with PBS and incubation for 24 h in DMEM. The cells were collected for protein extraction for a Western blot analysis.

### Statistical analyses

ImageJ (38) was used to quantify the densitometry of immunoblot bands. Data were analyzed using either the *t* test or a one-way ANOVA followed by Tukey's or Dunnett’s multiple comparisons tests, performed using the Prism 7 software program (GraphPad).

## Data availability

All data are contained within the article and available upon request.

## Conflict of interest

The authors declare that they have no conflicts of interest with the contents of this article.
